# Honeybees Learn Odour Mixtures via a Selection of Key Odorants

**DOI:** 10.1371/journal.pone.0009110

**Published:** 2010-02-08

**Authors:** Judith Reinhard, Michael Sinclair, Mandyam V. Srinivasan, Charles Claudianos

**Affiliations:** 1 Queensland Brain Institute, The University of Queensland, St. Lucia, Queensland, Australia; 2 School of Biomedical Sciences, The University of Queensland, St. Lucia, Queensland, Australia; 3 ARC Centre of Excellence in Vision Science, Australian National University, Canberra, Australian Capital Territory, Australia; 4 School of Information Technology and Electrical Engineering, The University of Queensland, St. Lucia, Queensland, Australia; Centre de Recherches su la Cognition Animale - Centre National de la Recherche Scientifique and Université Paul Sabatier, France

## Abstract

**Background:**

The honeybee has to detect, process and learn numerous complex odours from her natural environment on a daily basis. Most of these odours are floral scents, which are mixtures of dozens of different odorants. To date, it is still unclear how the bee brain unravels the complex information contained in scent mixtures.

**Methodology/Principal Findings:**

This study investigates learning of complex odour mixtures in honeybees using a simple olfactory conditioning procedure, the Proboscis-Extension-Reflex (PER) paradigm. Restrained honeybees were trained to three scent mixtures composed of 14 floral odorants each, and then tested with the individual odorants of each mixture. Bees did not respond to all odorants of a mixture equally: They responded well to a selection of key odorants, which were unique for each of the three scent mixtures. Bees showed less or very little response to the other odorants of the mixtures. The bees' response to mixtures composed of only the key odorants was as good as to the original mixtures of 14 odorants. A mixture composed of the other, non-key-odorants elicited a significantly lower response. Neither an odorant's volatility or molecular structure, nor learning efficiencies for individual odorants affected whether an odorant became a key odorant for a particular mixture. Odorant concentration had a positive effect, with odorants at high concentration likely to become key odorants.

**Conclusions/Significance:**

Our study suggests that the brain processes complex scent mixtures by predominantly learning information from selected key odorants. Our observations on key odorant learning lend significant support to previous work on olfactory learning and mixture processing in honeybees.

## Introduction

Olfaction is the primary sensory modality in most insects, including honeybees (*Apis mellifera* L.). Both in the darkness of the hive and in the outside environment bees encounter an overwhelming array of different scents. Honeybees are able to learn and discriminate between hundreds of odorants with exquisite sensitivity and specificity based on the odorants' carbon chain length, as well as type, position and number of functional groups [1], [2]. Physiological studies have explored the neural mechanisms underlying odour discrimination, showing that each odour triggers a specific spatio-temporal activity pattern in the first centre of olfactory processing of the bee brain, the antennal lobes (AL) [3]. Odour mixture learning has also been investigated extensively, both on a behavioural [4], [5], [6] and physiological level [7], [8]. However, most of these studies used mixtures composed of only a few odorants, even though the vast majority of scents that honeybees encounter in their natural environment are floral scents, which are complex blends of chemicals [9].

On average, a floral scent contains 20–60 different odorants [9]. The majority of floral odorants are terpenes or terpene derivatives, but there are also large numbers of alcohols, aldehydes, ketones, and esters. Different flower species emit different scents due to a difference in chemical composition, or in concentration and ratio of the components. A floral bouquet can even vary within a species, depending on the environmental conditions such as the location of an individual flower, the time of day, the pollination status, nectar content, and the age of the flower (rev. in [10]). Despite the complexity and variability of natural scents, honeybees display an amazing ability to learn, discriminate, and recognize floral odours.

It is still unclear exactly how bees perceive and interpret the information contained in complex scents. Do they learn a scent mixture as a unique configuration, or do they learn the individual odorants of the mixture as separate, equivalent elements? Both strategies of odour mixture learning have been investigated in the past (rev. in [11]). A particular series of studies [6], [12], [13], [14] reported that bees learnt the individual odorants of small scent mixtures as separate elements. Intriguingly, the authors showed that the bees did not respond to all of the individual odorants with the same efficiency after learning them as a mixture, suggesting that mixtures are interpreted through key components.

Based on these earlier studies, we now present a detailed behavioural investigation of odour mixture learning in honeybees using a simple olfactory conditioning procedure, the Proboscis-Extension-Reflex (PER) paradigm [15]. Our data show that bees learn a selection of mixture-specific key odorants as representatives for a scent mixture, suggesting that the brain filters incoming olfactory information from complex scents and only passes on information from selected odorants to higher brain centres.

## Results

### Odorant Learning Trials

**Figure 1:**
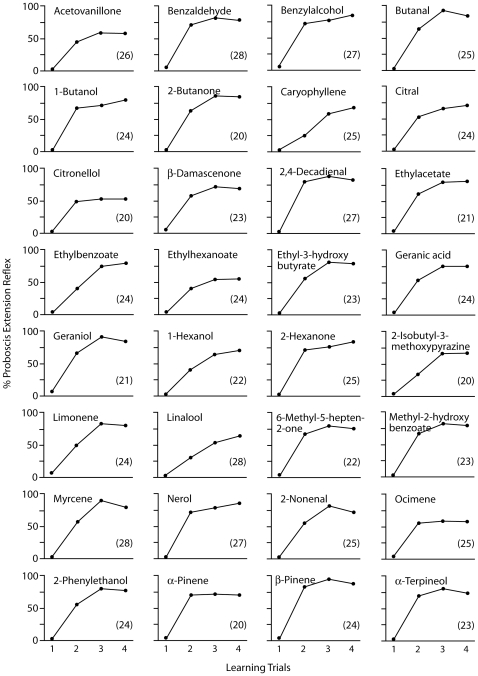
Acquisition Curves for 32 Floral Odorants. The ordinate represents the percentage of proboscis extensions (PER) to the training odorant. The abscissa indicates the training trials spaced over two days, three trials on day one and a fourth trial on day two. The numbers in brackets indicate the numbers of bees trained for each odorant. Odorants are listed alphabetically, for physico-chemical characteristics of odorants see Table 1.

Using the standard Proboscis-Extension-Reflex (PER) paradigm, we trained 32 groups of bees to 32 typical floral odorants (Table 1) [9], [16], [17]. Each group was trained to one odorant over two days, with three acquisition trials on day one and a fourth reinforcement trial on day two. The learning curves for each odorant are shown in Figure 1. The level of PER in the first trial was very low (between 0% and 9.5%). For the majority of odorants, the maximum PER response was recorded at trial 3, but no difference was found between the PERs recorded at trials 3 and 4 for any odorant (McNemar tests [2×2 Table]; in all cases p>0.05). The lowest maximum PER response for an odorant was recorded for citronellol (55.0%), and the highest maximum PER response was recorded for butanal (96.0%). Maximum PER responses for the other odorants were within this range. That is, all of the 32 odorants were learnt but not with the same efficiency. An overall (trial x odorant) General Linear Model Analysis of Variance (GLM ANOVA) showed a significant increase in responses along trials (*F(3,2202)* = 1127.7, p<0.001) and a significant heterogeneity among odorants (*F(31,734)* = 1.69, p = 0.012).

**Table 1:** Names and Characteristics of the Odorants Used.

No.	Odorant (Abbreviation)	Chemical Characteristics	Vapour Pressure (kPa at 25°C)	Density (g/mL at 25°C)	Purity	Company
1	Acetovanillone (AC)	Aromatic ketone	0.000002426	1.158	>98.0%	Aldrich
2	Benzaldehyde (BA)	Aromatic aldehyde	4.900	1.045	>99.5%	Aldrich
3	Benzylalcohol (BO)	Aromatic alcohol	0.0211	1.045	>99.0%	Sigma-Aldrich
4	Butanal (BUA)	Aldehyde	12.799	0.800	>99.0%	Fluka
5	1-Butanol (BUO)	Primary alcohol	1.1359	0.810	>99.9%	Sigma-Aldrich
6	2-Butanone (BUN)	Ketone	15.3321	0.805	>99.9%	Fluka
7	Caryophyllene (CY)	Sesquiterpene	0.0009	0.902	>98.5%	Sigma
8	Citral (CI)	Terpene aldehyde	0.0095	0.888	>95.0%	Fluka
9	Citronellol (CO)	Terpene alcohol	0.0130	0.857	>99.0%	Fluka
10	β-Damascenone (DA)	Norisoprenoid terpene	0.0007	0.800	95.0%	Aldrich
11	2,4-Decadienal (DE)	Unsaturated aldehyde	0.004	0.872	95.0%	Adrich
12	Ethylacetate (EA)	Ester	10.1	0.902	>99.9%	Fluka
13	Ethylbenzoate (EBZ)	Aromatic ester	0.024	1.045	>99.0%	Aldrich
14	Ethylhexanoate (EH)	Ester	0.2213	0.869	>99.0%	Aldrich
15	Ethyl-3-hydroxybutyrate (EHB)	Ester alcohol	0.0483	1.017	>98.0%	Aldrich
16	Geranic acid (GAC)	Terpene acid	0.0003	0.970	95.0%	Aldrich
17	Geraniol (GE)	Terpene alcohol	0.0030	0.879	>99.0%	Fluka
18	1-Hexanol (HO)	Primary alcohol	0.1263	0.814	>99.9%	Fluka
19	2-Hexanone (HN)	Ketone	1.7732	0.812	>99.5%	Fluka
20	2-Isobutyl-3-methoxypyrazine (IP)	Pyrazine	0.0364	0.990	99.0%	Aldrich
21	Limonene (LI)	Monoterpene	0.2053	0.842	>99.0%	Fluka
22	Linalool (LO)	Terpene alcohol	0.0121	0.870	>95.0%	Fluka
23	6-Methyl-5-hepten-2-one (MHN)	Unsaturated ketone	0.1707	0.855	99.0%	Aldrich
24	Methyl-2-hydroxybenzoate (MHB)	Aromatic ester	0.0093	1.174	>99.0%	Sigma-Aldrich
25	Myrcene (MY)	Monoterpene	0.3053	0.791	>95.0%	Fluka
26	Nerol (NE)	Terpene alcohol	0.0027	0.876	97.0%	Aldrich
27	2-Nonenal (NOA)	Unsaturated aldehyde	0.0038	0.846	97.0%	Aldrich
28	Ocimene (OC)	Monoterpene	0.1866	0.818	>90.0%	Aldrich
29	2-Phenylethanol (PHE)	Aromatic alcohol	0.0099	1.012	>98.0%	Fluka
30	α-Pinene (PIA)	Monoterpene	0.4653	0.858	98.0%	Aldrich
31	β-Pinene (PIB)	Monoterpene	0.32	0.866	>99.0%	Aldrich
32	α-Terpineol (TP)	Terpene alcohol	0.0038	0.930	90.0%	Aldrich

The odorants are listed alphabetically. Odorant vapour pressures and chemical characteristics such as functional groups are also given. Racemic mixtures were used in the case of odorants that had chiral carbons.

### Complex Scent Trials

**Figure 2:**
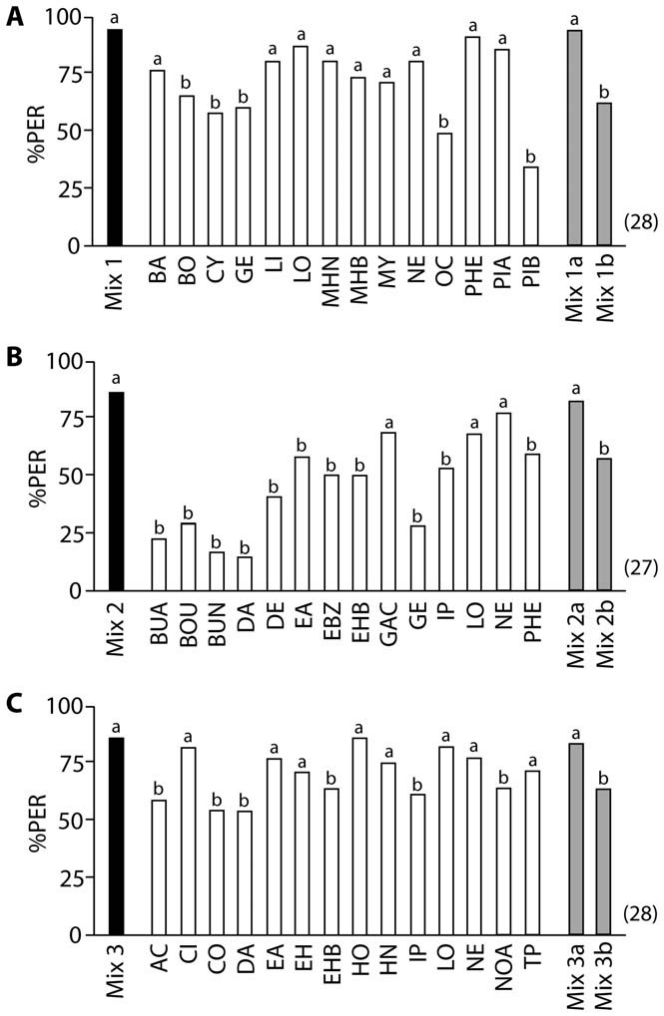
Key Odorant Signatures for Complex Scents. The ordinate represents the percentage of proboscis extensions (PER) to the training mixture (black bars), to the individual test odorants of which the mixture was composed (white bars), to a mixture of the key odorants (first grey bar), and to a mixture of non-key-odorants (second grey bar). The numbers in brackets indicate the numbers of bees trained and tested in each experiment. Different letters above bars (a or b) indicate significant differences between PER to the training mixture and to the individual odorants/key odorant mixture/non-key-odorant mixture (McNemar test [2×2 Table], Bonferroni corrected threshold p<0.0036). (A) Bees were trained to mixture 1, and responded well to nine key odorants; (B) bees were trained to mixture 2, and responded well to three key odorants; (C) bees were trained to mixture 3, and responded well to eight key odorants. For composition of the training mixture, key odorant mixture, and non-key-odorant mixture see Table 2. Odorants are listed alphabetically by their abbreviations; for corresponding odorant names, see Table 1.

Using the same PER paradigm as above, a group of bees was trained four times over two days with a complex scent mixture composed of 14 of the floral odorants (mixture 1, Table 2) (Table S1). The trained bees were then presented with each of the 14 single odorants (unrewarded), interspersed by rewarded training trials using the complex mixture. The PER responses to the single odorants (Fig. 2A) were compared to the PER response to the mixture as recorded at trial 4. The analysis showed that bees did not respond to all odorants equally (Cochran's Q Test, Q = 134.3, p<0.001); only nine odorants elicited PER responses at levels that were statistically indistinguishable from those elicited by the training mixture (McNemar tests [2×2 Table] with Bonferroni corrected threshold; in all cases p>0.0036): benzaldehyde, limonene, linalool, 6-methyl-5-hepten-2-one, methyl-2-hydroxybenzoate, myrcene, nerol, 2-phenylethanol, and α-pinene (Fig. 2A). These odorants were termed ‘key odorants’ for mixture 1. We then prepared a mixture of only these nine key odorants (mixture 1a, Table 2), as well as a mixture of the non-key-odorants (mixture 1b). A fresh group of bees was again trained to the original 14-odorant mixture (mixture 1), and then tested with the corresponding key odorant mixture and non-key-odorant mixture. There was no significant difference in PER level recorded between the full mixture and the key odorant mixture (McNemar test [2×2 Table] with Bonferroni corrected threshold, Chi square = 0; df = 1; p = 1.0), but bees responded significantly less to the non-key-odorant mixture (McNemar test [2×2 Table] with Bonferroni corrected threshold, Chi square = 8.1; df = 1; p<0.0036) (Fig 2A).

**Table 2:** Composition of Complex Odour Mixtures, Key Odorant Mixtures and Non-Key- Odorant Mixtures.

Complex Odour Mixtures		
Mixture 1	Mixture 2	Mixture 3
Benzaldehyde	Butanal	Acetovanillone
Benzylalcohol	1-Butanol	Citral
Caryophyllene	2-Butanone	Citronellol
Geraniol	β-Damascenone	β-Damascenone
Limonene	2,4-Decadienal	Ethylacetate
Linalool	Ethylacetate	Ethylhexanoate
6-Methyl-5-hepten-2-one	Ethylbenzoate	Ethyl-3-hydroxybutyrate
Methyl-2-hydroxybenzoate	Ethyl-3-hydroxybutyrate	1-Hexanol
Myrcene	Geranic acid	2-Hexanone
Nerol	Geraniol	2-Isobutyl-3-methoxypyrazine
Ocimene	2-Isobutyl-3-methoxypyrazine	Linalool
2-Phenylethanol	Linalool	Nerol
α-Pinene	Nerol	2-Nonenal
β-Pinene	2-Phenylethanol	α-Terpineol

Key Odorant Mixtures		
Mixture 1a	Mixture 2a	Mixture 3a
Benzaldehyde	Geranic acid	Citral
Limonene	Linalool	Ethylacetate
Linalool	Nerol	Ethylhexanoate
6-Methyl-5-hepten-2-one		1-Hexanol
Methyl-2-hydroxybenzoate		2-Hexanone
Myrcene		Linalool
Nerol		Neral
2-Phenylethanol		α-Terpineol
α-Pinene		

Non-Key-Odorant Mixtures		
Mixture 1b	Mixture 2b	Mixture 3b
Benzylalcohol	Butanal	Acetovanillone
Caryophyllene	1-Butanol	Citronellol
Geraniol	2-Butanone	β-Damascenone
Ocimene	β-Damascenone	Ethyl-3-hydroxybutyrate
β-Pinene	2,4-Decadienal	2-Isobutyl-3-methoxypyrazine
	Ethylacetate	2-Nonenal
	Ethylbenzoate	
	Ethyl-3-hydroxybutyrate	
	Geraniol	
	2-Isobutyl-3-methoxypyrazine	
	2-Phenylethanol	

The odorants in each mixture are listed alphabetically. Odorants were used in 1∶10 dilutions and mixed in 1∶1∶1 ratios. Key odorant mixtures and Non-key-odorant mixtures were composed based on the analysis of how bees learnt the corresponding complex mixtures, see text for details.

The entire experiment was repeated with a fresh group of bees and the second complex scent mixture, and then a third group of bees using the third scent mixture (Table 2). As in the first experiment, the trained bees did not respond equally to all of the single test odorants (Cochran's Q Test, Q = 163.6 and Q = 81.3, in both cases p<0.001), but only responded to some of them as well as to the respective training mixtures (Fig. 2B, C; McNemar tests [2×2 Table] with Bonferroni corrected threshold; in all cases of equivalent response p>0.0036). When trained with mixture 2, bees responded well to three of the single odorants: geranic acid, linalool, and nerol (Fig. 2B). When trained with mixture 3, bees responded well to eight of the single odorants: citral, ethylacetate, ethylhexanoate, 1-hexanol, 2-hexanone, linalool, nerol and α-terpineol (Fig. 2C). Key odorant mixtures and non-key-odorant mixtures for mixture 2 and 3 were prepared based on this analysis (Table 2, mixture 2a, 2b, mixture 3a, 3b), and new groups of bees were trained first to the original 14-odorant mixtures and then tested with the corresponding key odorant and non-key-odorant mixtures. There was no significant difference in PER level recorded between a complex mixture and its corresponding key odorant mixture (McNemar test [2×2 Table] with Bonferroni corrected threshold; in both cases p>0.0036), but bees responded significantly less to the non-key-odorant mixtures (McNemar test [2×2 Table] with Bonferroni corrected threshold; Chi square = 9.1 (mix 2b) and 7.1 (mix 3b); df = 1; in both cases p<0.0036) (Fig. 2B, C). In summary, after training to a complex mixture bees responded selectively better to some odorants (termed ‘key odorants’), with significantly lower response to the other odorants of a mixture.

Whether an odorant became a key odorant was not linked to the odorant's chemical structure (functional group) or volatility. There were straight chain as well as aromatic molecules, aldehydes, terpenes, alcohols, ketones, esters and an acid among the key odorants, and their vapour pressures ranged from 0.0003 to 10.1 kPA (Table 3). Whether an odorant became a key odorant in a mixture was not correlated to the efficiency with which it was learnt on its own (Figure S1). When comparing the maximum PER recorded for an odorant during acquisition trials with the PER recorded when the odorant was learnt as part of the complex mixture, we found that, for example, butanal was learnt very well on its own (96.0% PER), but only 21.4% of bees responded to butanal, when it was learnt as part of mixture 2 (Table 4). On the other hand, linalool had a rather mediocre learning efficiency of 64.3% when presented on its own, but when the bees learnt it as part of mixture 1, 86.4% of bees responded to it. When linalool was learnt as part of mixture 2, only 66.7% of bees responded, but as part of mixture 3, the PER for linalool was again high at 81.8% (Table 4). There was no general pattern, that is an odorant could be learnt well on its own, but not well as part of a mixture, or the opposite could be the case. A good learning efficiency was no indicator for an odorant becoming a key odorant.

**Table 3:** Physico-chemical Characteristics of Key Odorants.

Key Odorants of Mixture 1	Chemical Characteristics	Vapour Pressure (kPa; 25°C)
Benzaldehyde	Aromatic aldehyde	4.900
Limonene	Monoterpene	0.2053
Linalool	Terpene alcohol	0.0121
6-Methyl-5-hepten-2-one	Unsaturated ketone	0.1707
Methyl-2-hydroxybenzoate	Aromatic ester	0.0093
Myrcene	Monoterpene	0.3053
Nerol	Terpene alcohol	0.0027
2-Phenylethanol	Aromatic alcohol	0.0099
α-Pinene	Monoterpene	0.4653

Key Odorants of Mixture 2	Chemical Characteristics	Vapour Pressure (kPa; 25°C)
Geranic acid	Terpene acid	0.0003
Linalool	Terpene alcohol	0.0121
Nerol	Terpene alcohol	0.0027

Key Odorants of Mixture 3	Chemical Characteristics	Vapour Pressure (kPa; 25°C)
Citral	Terpene aldehyde	0.0095
Ethylacetate	Ester	10.1
Ethylhexanoate	Ester	0.2213
1-Hexanol	Primary alcohol	0.1263
2-Hexanone	Ketone	1.7732
Linalool	Terpene alcohol	0.0121
Nerol	Terpene alcohol	0.0027
α-Terpineol	Terpene alcohol	0.0038

The key odorants are listed alphabetically for each mixture. Composition of original mixtures 1, 2, and 3 see Table 2.

**Table 4:** Maximum PER Response [%] to Odorants Relative to Occurrence in Mixtures.

No.	Odorant	Alone	Mix 1	Mix 2	Mix 3
1	Acetovanillone	61.5	-	-	57.7
2	Benzaldehyde	83.3	76.0	-	-
3	Benzylalcohol	86.7	65.4	-	-
4	Butanal	96.0	-	21.4	-
5	1-Butanol	79.2	-	28.6	-
6	2-Butanone	90.0	-	17.4	-
7	Caryophyllene	66.7	57.1	-	-
8	Citral	70.8	-	-	81.8
9	Citronellol	55.0	-	-	56.0
10	β-Damascenone	73.9	-	15.0	55.0
11	2,4-Decadienal	88.9	-	40.9	-
12	Ethylacetate	80.9	-	61.9	76.2
13	Ethylbenzoate	79.2	-	50.0	-
14	Ethylhexanoate	54.2	-	-	71.4
15	Ethyl-3-hydroxybutyrate	82.6	-	50.0	63.6
16	Geranic acid	75.0	-	68.2	-
17	Geraniol	90.5	60.0	28.6	-
18	1-Hexanol	68.2	-	-	85.7
19	2-Hexanone	84.0	-	-	75.0
20	2-Isobutyl-3-methoxypyrazine	65.0	-	54.6	61.9
21	Limonene	83.3	80.9	-	-
22	Linalool	64.3	86.4	66.7	81.8
23	6-Methyl-5-hepten-2-one	77.3	80.8	-	-
24	Methyl-2-hydroxybenzoate	86.9	73.9	-	-
25	Myrcene	89.3	71.4	-	-
26	Nerol	85.2	78.3	76.2	76.9
27	2-Nonenal	84.0	-	-	66.7
28	Ocimene	60.0	48.8	-	-
29	2-Phenylethanol	79.2	92.0	58.3	-
30	α-Pinene	71.3	83.3	-	-
31	β-Pinene	95.8	33.3	-	-
32	α-Terpineol	82.6	-	-	72.7

The odorants are listed alphabetically. Given are the percentages of maximum Proboscis-Extension-Reflex (PER) response to each odorant (*N* = 20–28 bees), depending on whether the odorant was learnt when presented on its own (Alone) or learnt when presented as part of mixture 1 (Mix 1), mixture 2 (Mix 2) or mixture 3 (Mix 3). For mixture composition see Table 2. -: Odorant was not part of the mixture.

To investigate discrimination and generalisation, respectively, of the three 14-odorant mixtures, a fresh group of bees was trained to mixture 1 (Table 2), and then tested with mixtures 2 and 3. This was repeated by training a new group of bees to mixture 2 and testing them with mixtures 1 and 3, and training a third group of bees to mixture 3 and testing them with mixtures 1 and 2. Discrimination of the three mixtures was generally poor, that is bees generalized across mixtures, likely due to the mixtures sharing a number of odorants including key odorants (Table 5). When bees were trained to mixture 1, they clearly generalized to mixture 2, which shared four odorants with mixture 1 including three of the mixture-1 key odorants, namely linalool, nerol, and 2-phenylethanol. Mixture 3, which shared only two odorants with mixture 1 (both of them mixture-1 key odorants: linalool and nerol), was generalized by only 50.0% of the bees (McNemar test [2×2 Table] Chi square = 7.11; df = 1; p = 0.008). When trained to mixture 2 (three key odorants), the bees generalized to both mixture 1 and mixture 3. Both test mixtures contained two of the mixture-2 key odorants (linalool and nerol), and mixture 1 shared all up four odorants with mixture 2, while mixture 3 even shared 6 odorants with mixture 2. When trained to mixture 3 (eight key odorants), bees showed some discrimination of mixture 1, which had two odorants in common with mixture 3, both of them mixture-3 key odorants, namely linalool and nerol (61.5% PER; McNemar test [2×2 Table] Chi square = 6.13; df = 1; p = 0.013). However, they generalized to mixture 2, which shared six odorants with mixture 3 including three of the mixture-3 key odorants (ethylacetate, linalool, nerol). In summary, bees generalized well between the complex mixtures, although the testing mixtures contained in each case only two or three training mixture key odorants. This suggests that detection of key odorants is crucial for generalising across mixtures, and that the minor, non-key odorants do not play an important role in mixture discrimination.

**Table 5:** Discrimination of Complex Odour Mixtures: PER Response [%].

Training Mixtures	Testing Mixtures		
	Mix 1	Mix 2	Mix 3
**Mix 1 (9)**	92.5 (9)	90.0 (3)	50.0 (2)*
**Mix 2 (3)**	86.4 (2)	88.6 (3)	81.8 (2)
**Mix 3 (8)**	61.5 (2)*	96.2 (3)	94.3 (8)

Table shows PER response in % to the training and test mixtures (*N* = 27 bees for each trial). Numbers in brackets signify number of key odorants in training mixtures, and numbers of odorants in the test mixture that are also key odorants of the training mixture. *: Significant difference in PER response between testing mixture and training mixture (grey boxes), McNemar Chi square test [2×2 Table], p<0.05. For mixture composition see Table 2.

### Odorant Uniqueness Trials

**Figure 3:**
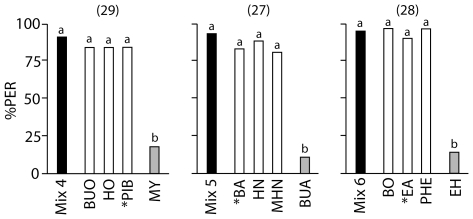
Effect of Odorant Uniqueness on Key Odorant Signatures. The ordinate represents the percentage of proboscis extensions (PER) to the training mixture (black bars), to the individual test odorants that the mixture was composed of (white bars), and to a control odorant that was not part of the training mixture (grey bars). Each mixture was composed of two odorants with the same functional group and one unique odorant with a different functional group, indicated by *. The control odorant shared the chemical characteristic of the unique odorant. The numbers in brackets indicate the numbers of bees trained and tested in each experiment. Different letters above bars (a or b) indicate significant differences between PER to the training mixture and to the individual odorants (McNemar test [2×2 Table], Bonferroni corrected threshold p<0.0125). Bees responded to the individual odorants of a mixture as well as to the training mixture, but responded significantly less to the control odorant. (Left) Bees were trained to mixture 4; (Centre) bees were trained to mixture 5; (Right) bees were trained to mixture 6. For mixture compositions see Table 6. Odorants are listed alphabetically by their abbreviations; for corresponding odorant names, see Table 1.

We next investigated whether chemical distinctiveness of an odorant within a mixture has an effect on how key odorants are determined. The rationale behind this experiment is based on the hypothesis that a distinctive odorant can give a mixture its specific character. We composed a mixture of three odorants, with one of the odorants having a different functional group than the other two: β-pinene (terpene), 1-butanol and 1-hexanol (alcohols) (mixture 4, Table 6). Bees were trained as above four times over two days with the mixture, and then tested with the three single odorants (unrewarded), as well as myrcene as a control odorant that shared a chemical characteristic with the “unique” odorant, but that was not part of the training mixture. Responses to the test odorants were all at the same level and indistinguishable from the training mixture (Fig. 3 left; Cochran's Q Test, Q = 6.0, p = 0.112; McNemar test [2×2 Table] with Bonferroni corrected threshold; in all cases p>0.0125), that is the bees did not pick specific key odorants for mixture 4. However, the bees showed significantly decreased PER to the control odorant myrcene (McNemar test [2×2 Table] Chi square = 16.1; df = 1; p<0.001), showing that the bees did not just respond to any odorant, but that they had indeed learnt the three odorants of the mixture.

**Table 6:** Composition of Odour Mixtures for Odorant Uniqueness and Odorant Concentration Tests.

Uniqueness Tests	Concentration Tests
**Mixture 4**	**Mixture 7**
1-Butanol	Limonene 1∶10
1-Hexanol	Myrcene 1∶100
β-Pinene*	β-Pinene 1∶100
**Mixture 5**	**Mixture 8**
Benzaldehyde*	Limonene 1∶100
2-Hexanone	Myrcene 1∶10
6-Methyl-5-hepten-2-one	β-Pinene 1∶100
**Mixture 6**	**Mixture 9**
Benzylalcohol	Limonene 1∶100
Ethylacetate*	Myrcene 1∶100
1-Phenylethanol	β-Pinene 1∶10
	**Mixture 10 (Control)**
	Limonene 1∶10
	Myrcene 1∶10
	β-Pinene 1∶10
	**Mixture 11 (Control)**
	Limonene 1∶100
	Myrcene 1∶100
	β-Pinene 1∶100

The odorants in each mixture are listed alphabetically. Odorants were used in 1∶10 dilutions unless otherwise specified, and mixed in 1∶1∶1 ratios. *: Chemically unique odorant in the mixture with respect to functional group.

The same was true, when the experiment was repeated with fresh groups of bees and two more mixtures of three odorants each: benzaldehyde (aldehyde), 2-hexanone and 6-methyl-5-hepten-2-one (ketones) with butanal as control odorant (mixture 5, Table 6, Fig. 3 centre); ethylacetate (ester), benzylalcohol and 2-phenylethanol (alcohols) with ethyl hexanoate as control odorant (mixture 6, Table 6, Fig. 3 right). The bees responded equally to all training odorants (Cochran's Q Test, Q = 8.1, p = 0.07; and Q = 6.0, p = 0.112; McNemar test [2×2 Table] with Bonferroni corrected threshold; in all cases p>0.0125), but responded significantly less to the respective control odorant (McNemar test [2×2 Table] Chi square = 9.1, df = 1, p = 0.002; and Chi square = 13.1, df = 1, p<0.001). In summary, chemical distinctiveness of an odorant within a mixture did not seem to play a role in determining key odorants.

### Odorant Concentration Trials

Nine groups of 30 bees were trained four times over two days as above, to limonene, myrcene and β-pinene, at three different concentrations each (1∶10, 1∶100, and 1∶1000), to establish the acquisition efficiency for these odorants at different concentrations. All three odorants were learnt well at all concentrations, with the maximum PER recorded at 86.7% for limonene, 81.5% for myrcene, and 87.5% for β-pinene (Table S2). Acquisition speed was slightly slower at lower concentrations (1∶1000, 1∶100) with the maximum PER reached by trial 4, while at the high concentration (1∶10) the maximum PER was reached by trial 3. To test whether the bees could discriminate the three fairly similar floral odorants, discrimination trials at all three concentrations were conducted. The bees had no problem discriminating the three odorants at all three concentrations (Table S3; McNemar test [2×2 Table] with Bonferroni corrected threshold; in all cases p<0.001), with discrimination better at higher concentrations, a result previously also reported by [18].

**Figure 4:**
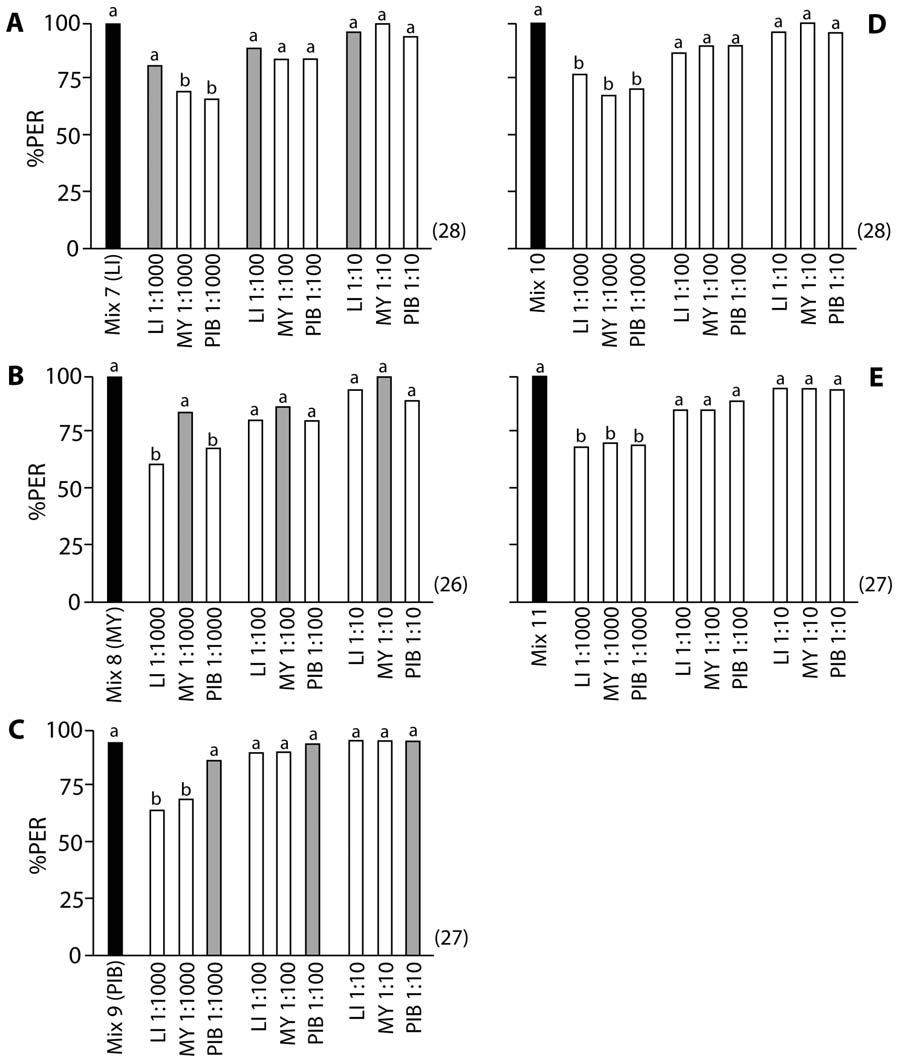
Effect of Odorant Concentration on Key Odorant Signatures. The ordinate represents the percentage of proboscis extensions (PER) to the training mixture (black bars), and to different concentrations of the individual test odorants that the mixture was composed of (grey and white bars). Training mixtures were composed of the same three odorants, but with one odorant at higher concentration than the other two. Grey bars indicate response to the odorant that was present at the high concentration in the respective training mixture. (A) Bees were trained to mixture 7, which contained LI at 1∶10; (B) bees were trained to mixture 8, which contained MY at 1∶10; (C) bees were trained to mixture 9, which contained PIB at 1∶10; (D) bees were trained to control mixture 10, which contained all odorants at 1∶100; (E) bees were trained to control mixture 11, which contained all odorants at 1∶10. The numbers in brackets indicate the numbers of bees trained and tested in each experiment. Different letters above bars indicate significant differences between PER to the training mixture and to the individual odorants (McNemar test [2×2 Table], Bonferroni corrected threshold p<0.0056). For mixture compositions see Table 6. Odorants are listed alphabetically by their abbreviations; for corresponding odorant names, see Table 1.

To test whether the concentration of an odorant within a mixture has an effect on how key odorants are determined, we composed three mixtures of the above three odorants, each having one odorant at a higher concentration than the other two (Table 6). A group of bees was trained to mixture 7, which had limonene at a high concentration, and myrcene and β-pinene at lower concentrations. The bees were then tested with the single unrewarded odorants each at 1∶1000, 1∶100, and 1∶10 concentration. Significant differences in PER responses to the single odorants were detected (Cochran's Q Test, Q = 53.8, p<0.001). At the low testing concentration (1∶1000), the bees responded to limonene as well as to the training mixture (McNemar test [2×2 Table] with Bonferroni corrected threshold; Chi square = 0.25; df = 1; p = 0.617), but significantly less to myrcene (p = 0.004) and β-pinene (p = 0.003). However this effect was lost at the higher testing concentrations when bees responded to all three odorants as well as to the training mixture (Fig. 4A; McNemar test [2×2 Table] with Bonferroni corrected threshold; in all cases p>0.0056). The experiment was repeated with a second group of bees trained to mixture 8 (myrcene at high concentration), and with a third group of bees trained to mixture 9 (β-pinene at high concentration), and tested in the same way. When tested with mixture 8, the bees responded well only to myrcene in the tests when compared to the training mixture (Fig. 4B; McNemar test [2×2 Table] with Bonferroni corrected threshold; Chi square = 0.57; df = 1; p = 0.449; preceding Cochran's Q Test, Q = 51.9, p<0.001), and when trained with mixture 9, they responded well only to β-pinene when compared to the training mixture (Fig. 4C; McNemar test [2×2 Table] with Bonferroni corrected threshold; Chi square = 0; df = 1; p = 1.0; preceding Cochran's Q Test, Q = 52.2, p<0.001), but again this effect only existed at the low testing concentration. At higher testing concentrations the bees responded to all test odorants with the same intensity as to the training mixture (McNemar test [2×2 Table] with Bonferroni corrected threshold; in all cases p>0.0056).

Two final groups of bees were trained and tested as above using two control mixtures, which contained the three odorants either all at 1∶100 or all at 1∶10 concentration (mixtures 10 and 11, Table 6). Now the bees responded equally well to all three test odorants at any one concentration, with only the lowest test concentrations of odorants resulting in a significantly lower PER than that recorded for the corresponding training mixture (Fig. 4D, E; McNemar test [2×2 Table] with Bonferroni corrected threshold; in all cases p<0.0056). Taken together, these results demonstrate that the concentration of an odorant within a mixture has a positive effect on this odorant becoming a key odorant, however the effect is only evident at low testing concentrations.

## Discussion

The sense of smell plays a vital role in all animal species in detecting and processing information regarding virtually every aspect of life, from reproduction to foraging, from navigation to detecting danger and disease. After decades of research into olfactory processing, we are now beginning to understand how the brain deciphers the multifaceted information contained in complex natural scents. Here, we present a detailed behavioural study on learning of complex odours, using an exquisite olfactory model organism, the honeybee, and a simple olfactory conditioning procedure, the Proboscis-Extension-Reflex (PER) paradigm. We show that bees trained to scent mixtures of 14 odorants do not respond to all odorants of a mixture equally when the individual odorants are presented on their own. Rather, the bees predominantly learn a selection of key odorants as representatives for each mixture, with the number and type of key odorants depending on the mixture composition. Mixtures composed of only the key odorants could not be distinguished from the original training mixtures composed of 14 odorants, while the bees responded significantly less to mixtures composed of the other, non-key-odorants of the training mixtures.

The fact that bees responded to some odorants but not to others was not because the bees lacked the ability to learn the odorants. Our initial odorant acquisition experiments demonstrated clearly that all of the used odorants were learnt by the bees. This suggests that bees have olfactory receptors (ORs) that respond to the tested odorants, which is not surprising considering that we used common floral odorants [9], [16], [17]. Importantly, there was no all-or-nothing response to the single odorants after the bees were trained to a mixture, but rather a graded response. One could argue that in some cases the difference in response between a key odorant and a non-key-odorant was so small that making a distinction between the two seems artificial. However, in most cases the differences in response were obvious and highly significant, with some odorants clearly standing out. Also, taking our results on odorant concentration into account, it is possible that the difference in response between key odorants and non-key-odorants would be much greater, if they were present in different concentrations within the mixture as is the case in natural floral scents. We have therefore interpreted our results in line with previous studies, which described the same phenomenon and where the authors came to the conclusion that bees learn “key components” from complex odours [5], [6], [12], [13], [14].

Recent work on complex odour processing in the moth *Manduca sexta* reports strikingly similar results [19], [20]. The floral scent of the moth's food plant *Datura wrightii* emits>60 different compounds, but only nine out of these elicited robust neural responses. Behavioural experiments further showed that only three of these nine odorants were necessary and sufficient to elicit flower-foraging and feeding behaviours [20]. If these three odorants were removed from the floral mixture, then behavioural responses were depressed to control levels. However, and in contrast to our study the odorants were only effective in eliciting full foraging behaviour in moths as a mixture. This difference might be due to the fact that *M. sexta* is a specialist feeder [21], [22] requiring the presence of at least these three key odorants to identify the correct plant. The fact that honeybees, which are generalist feeders, respond not only to the mixture of key odorants, but also to the single key odorants, might be a reflection of their ability for broader odour generalisation.

### The Key Odorant Hypothesis

A number of previous studies have investigated whether honeybees learn scent mixtures as a unique configuration, or whether they learn the individual odorants of a mixture as separate, equivalent elements (rev. in [11]). We believe that the key odorant hypothesis encompasses features of both. Bees learn and respond to all the individual elements of our mixtures, albeit not to the same extent, suggesting that some odorants (key odorants) are more representative of the mixture than others (non-key-odorants). Although statistically indistinguishable, in most cases the single key odorants do not trigger a behavioural response at quite the same level as the full mixture, or as the key odorant mixture. This suggests that the mixture of key odorants in itself might be perceived as a configural stimulus.

Our observations on key odorant learning lend significant support to a number of interesting phenomena reported in previous work on olfactory learning and mixture processing in honeybees. For example, a study on learning of binary mixtures described that bees show lowered PER to odorants A or B after conditioning to a mixture of A and B, compared to equivalent conditioning to the pure odorants [4]. However, this overshadowing effect was not consistent, but depended on the mixture of odorants [23]. In some binary mixtures overshadowing occurred, in others it did not – which corresponds to our own observations of an odorant being a key odorant in one mixture but not in another. Our results are also in line with the above mentioned studies [5], [6], [12], [13], [14], which all report that bees trained with mixtures of odorants respond to some of the single odorants better than to others. In these studies the authors also noted that the olfactory background, i.e. the mixture composition, affected how the individual odorants were learnt. For example, an odorant that was learnt poorly on its own appeared to be an active component after being learned in a complex mixture [6]. Again, our own results correspond to those reported. The authors interpreted their data as a potentiation effect, that is an odorant could be potentiated by the presence of other odorants in the mixture.

### What Makes a Key Odorant?

The most burning question is: what determines whether an odorant in a mixture becomes a key odorant or not? Our results suggest that the number and type of key odorants depend on the mixture. Some odorants were key odorants for all three mixtures, others only for one or two. That is, if an odorant was key odorant for one mixture it was not necessarily a key odorant for another mixture. It has been reported that functional groups can play a role in odorant learning [24], but we found no evidence that the functional group of an odorant had an effect on it becoming a key odorant or not. Odorants of any molecular structure could become key odorants, and a highly volatile odorant such as benzaldehyde or ethylacetate, had as much chance of becoming a key odorant as an odorant of low volatility, such as nerol or geranic acid. Surprisingly, not even the learning efficiency of an odorant played a role. One would assume that an odorant bees learn well has a higher likelihood of being picked as key odorant for a mixture, however our results do not support this assumption. An odorant could be learnt very well or very badly on its own and both become or not become a key odorant for a mixture – depending entirely on the composition of the mixture. Similar observations were reported by [6].

It occurred to us that bees might choose the one odorant of a mixture that is chemically most distinct and thus characterises a mixture best – similar to studies by [25], showing that odorant salience can play a role in odorant learning and discrimination. However, our experiments revealed that bees did not give preference to chemically unique odorants when picking key odorants. The only effect on key odorant learning that we found was linked to odorant concentration. If an odorant was present at high concentration in a mixture, it became a key odorant, although this was only obvious when the test odorants were presented at low concentrations. At higher testing concentrations bees responded to all odorants equally. Apart from concentration, we could not identify any specific factors that determined whether an odorant became key odorant; it seemed to depend entirely on the mixture. Of course, there must be neural and molecular mechanisms in place that actually determine whether an odorant becomes key odorant for a mixture, however at this stage we can only speculate as to these mechanisms (see also below).

We made an interesting side observation with respect to odorant number in a mixture. When trained to complex mixtures composed of 14 odorants, the bees responded better to a subset of key odorants (between 3 and 9 depending on mixture), while in smaller mixtures composed of only three odorants, bees responded to all of them equally. This corresponds to earlier studies, showing that when trained to simple mixtures bees respond to most of the odorants to some extent [5], [6], but only respond to subsets of 6 odorants when trained to a complex floral fragrance [14]. It is possible that this is due to the specific mixture compositions and concentrations used, and in other small mixtures bees might choose only one or two as key odorants. However, it seems plausible that bees use all information available when presented with small mixtures, but focus on a few key odorants when presented with more complex mixtures.

### What Are the Mechanisms Underlying Key Odorant Learning?

At this stage we can only speculate as to the mechanisms underlying key odorant learning. Many studies have suggested that the wiring and neural interactions in the first olfactory neuropil (antennal lobe, AL) between incoming olfactory sensory neurons (OSN), local interneurons (LN) and outgoing projection neurons (PN) significantly affects odour processing [7], [8], [11], [26], [27], [28], [29]. It is therefore likely that the mechanisms underlying key odorant learning are part of the complex processing machinery occurring in the AL glomeruli, such as PN-LN-PN lateral inhibition, LN-PN excitation, LN-OSN-PN inhibition and disinhibition, to mention just a few examples [11], [26]. In addition, feedback from the higher-order processing centres such as mushroom bodies (MBs) and protocerebrum to the AL (reviewed in [30]) could have modulatory effects on the AL circuits that might be involved in key odorant processing. Clearly, unraveling the neural mechanisms in the AL potentially underlying key odorant learning will be a major challenge.

A recent study on floral scent coding in moths [19] suggests that the AL is indeed involved in key odorant processing: only 9 out of the 60 odorants from the floral scent mixture tested elicited robust neural responses in the moth AL. Several studies of mixture processing using calcium imaging of the honeybee and *Drosophila* AL (rev. in [11]), provide further evidence. Glomerular activity patterns evoked by mixtures are generally not merely a summation of the responses evoked by the individual components, suggesting that mixtures are processed based on inhibitory/excitatory circuits in the AL [7], [8], [26]. Mixture-evoked responses most often are reduced compared to the responses to individual odorants, although this is glomerulus-dependent and mixture synergism exists as well. Importantly mixture-evoked patterns are always more similar to the patterns evoked by salient (stronger) odorants [8].

On a molecular level it would be worth investigating to what extent the overlapping response spectra of the olfactory receptors affect key odorant learning. Each OSN carries one specific type of 7-transmembrane olfactory receptor protein (OR), and OSNs with the same OR terminate in the same glomerulus in the AL [31], [32]. Importantly, most ORs are broadly tuned responding to a range of chemically similar odorants [33]. When a honeybee detects a scent mixture, different ORs respond to the different odorants, but due to their presumed broad tuning one OR might respond to several odorants, with the level of response depending on odorant concentration. It is conceivable that the specific combinations and concentrations of odorants within a mixture could have a significant effect on the OSN activation levels via overlapping OR response spectra, thus influencing key odorant processing in the AL circuits. As with the neural circuits, unravelling these potential molecular mechanisms poses a major challenge.

### Biological Significance of Key Odorant Learning

The complex natural scents that bees encounter in their environment are mostly floral of origin and highly variable. No two floral scents are the same, and even between two visits to the same flower the aroma can change due to depletion of pollen and nectar, or visits by other pollinators (rev. in [10]). To identify a rewarding flower and repeatedly return to it in-spite of its changing bouquet, and at the same time avoid visiting unrewarding or depleted flowers that emit a similar bouquet, honeybees have to achieve a perfect balance between scent discrimination and scent generalization. Key odorant learning provides an efficient strategy to achieve this balance. It provides bees with a unique selection of odorants, sufficient to discriminate where necessary and generalize where possible. For example mixture 1 contained nine key odorants (Table 5). If the bee encounters a scent that contains three of these key odorants (e.g. mixture 2), she generalizes, but if the novel scent has only two of the key odorants (e.g. mixture 3), she discriminates. In this case more than two key odorants were needed to ensure generalization. We would expect generalization to be even better, if the mixtures shared more than three key odorants. It is of course possible that the other, non-key-odorants contributed to generalization as well, with mixture 1 and 2 sharing four odorants all up, while mixture 1 and 3 only share two odorants. In contrast, if a scent contained only three key odorants (mixture 2), any scent that contained two out of these three was considered similar enough (Table 5). Again, it is possible that the shared non-key-odorants contributed to generalization.

Whether a bee generalizes or discriminates between complex odours would of course also depend on the concentration of the odorants in the mixtures, which was not tested here. However, it is intriguing that sharing only a few odorants was sufficient to generalize between these complex mixtures, irrespective of the number of other, non-key-odorants in the test mixtures that differed. This suggests that the key odorants are indeed a bee's “focus” when detecting and processing a floral scent, and information from non-key-odorants might be of much less relevance for odour discrimination and generalization.

It is important to remember that in our studies we used 1∶1 ratios of all odorants for the complex mixtures, while in nature odorants occur at different ratios within a mixture. Varying concentrations and ratios of odorants contribute to scent recognition and discrimination [34]. For example, two related flower species can contain the same odorants but at different ratios, resulting in slightly different key odorant signatures, which will assist bees in discriminating between the two flower species. Even the same flower can produce different ratios of odorants in its bouquet throughout the day depending on its circadian rhythm or the amount of nectar or pollen available [35]. If a bee has learnt the key odorant signature for a flower while it was producing nectar, and then returns to it when the flower has been depleted, the bee encounters a quantitatively different odorant profile, with for example some former key odorants now at low concentration. The changed odorant ratio will tell the bee that even though the flower looks exactly the same, its nectar production has changed and it is no longer worth visiting.

### Conclusion

Across the animal kingdom, chemosensory systems are remarkably similar in design, sharing a number of fundamental mechanisms [36], [37]. It is likely that key odorant processing of scents is not unique for honeybees or even insects, but is used by all animals. A study on human subjects showed that, just like bees, humans could only identify a limited number of odorants within mixtures [38], suggesting that key odorant learning is also a trait of the human brain.

Two issues have to be kept in mind: (1) Experience can play an important role in the way odours are processed [39], [40]. Conditioning can modify AL activity and improve separation of odour representations. This suggests that learning leads to “tuning” of the AL such that relevant odours become more discriminable [41]. It is therefore possible that key odorant signatures change over time depending on individual scent experiences. It is even conceivable that scent mixtures are only learnt via key odorants when they are novel, but with continued experience with the same scent, the initial key odorant signature might increasingly become a configural stimulus. (2) It is also possible that key odorant signatures differ depending on context. For example the key odorant signatures that the bees learnt in our design may be different if the bees were trained differentially, that is if one mixture was rewarded and the other punished. The bees then may learn key odorant signatures that enable them to discriminate between the mixtures.

Apart from unraveling the neural circuits in the AL and the molecular mechanisms that potentially underlie key odorant learning, future research needs to focus on the plasticity of scent learning, in particular the role of environmental and individual scent experience in modulating key odorant signatures and scent perception.

## Materials and Methods

### Insects

For each experiment 30 honeybees were captured at the entrance of an outdoor hive and were cooled in a refrigerator for 10 min until they stopped moving. Then they were harnessed in Eppendorf vials that had the lid and tip removed to create a tapered tube. The bees were positioned so that their heads protruded through the narrow opening, and their mouthparts and antennae could move freely. They were fixed with a thin strip of fabric tape behind the head, and supported with some cotton wool underneath the abdomen filling the hollow of the Eppendorf vial. Vials were fixed in rows on a Perspex rack. Harnessed bees were left for 2 h in a bee humidor in the dark (26°C, 55% RH) to adjust. Ten minutes before commencing the experiments, each bee was checked for intact PER by touching one antenna with a toothpick that had been dipped into 1M sucrose solution without allowing feeding. Only bees that extended their proboscis in response to the sugar stimulus were used for the experiments, the others were discarded (approx. 3% of the bees).

### Odour Delivery

The odours were delivered by a custom-built olfactory stimulus controller, which allowed presentation of a constant clean airstream and an odorant through separate channels. Pressurised purified air was directed into the stimulus controller, and a constant clean airstream of 800ml/min was delivered through the first channel via Teflon tubing connected to a 1ml syringe containing a clean filter paper strip. The first channel carrying the clean airstream remained open at all times. Odours were delivered through the second channel via Teflon tubing connected to a 1ml syringe that contained a filter paper strip with the odour applied. The syringes were mounted next to each other with their tips approximately 1.5cm distant from the bee head. The second channel was only opened for odour delivery for a 6-s interval via an electronic valve controlled remotely by the experimenter. Importantly, opening of the second channel did not change the overall volume of the airflow, as the airstream was split equally and without delay between the first and second channel for the interval of odour delivery. After the 6-s stimulation, the second channel was closed and the airstream again directed only through the first channel (clean air) until the next stimulation. An extraction fan placed behind the bee was used to remove the odour from the experimental area and avoid lingering of any odour traces.

### Odours

Thirty-two floral odorants (Sigma Aldrich, Australia) were used as training and testing stimuli (see Table 1). Racemic mixtures were used in the case of molecules that had chiral carbons. Pure odorants were diluted 1∶10, 1∶100, and 1∶1000 in Ethanol (acetovanillone and benzylalcohol) or in hexane (all other odorants). For the complex scent experiment three mixtures were created, each containing 1∶1 ratios of 14 odorants at 1∶10 dilution (Table 2 top). The composition of the three mixtures including overlap in odorants was determined in a semi-random fashion, ensuring that each of the 32 odorants was represented at least once and that any two mixtures overlapped in at least 2 and no more than 6 odorants. Six more mixtures were created after testing, three of which were composed of the odorants to which bees had responded well (key odorant mixtures, Table 2 centre) (see below and results for details). The other three mixtures were composed of the odorants to which bees had not responded well (non-key-odorant mixtures, Table 2 bottom).

For the chemical uniqueness experiments, three mixtures of odorants (1∶10 dilutions, 1∶1∶1 ratio) were created, with one of the odorants having a different functional group than the other two: β-pinene/1-butanol/1-hexanol; benzaldehyde/2-hexanone/6-methyl-5-hepten-2-one; ethylacetate/benzylalcohol/2-phenylethanol (Table 6). For the concentration experiments, three mixtures of limonene, myrcene and β-pinene (1∶1∶1 ratio) were created containing one of the odorants at 1∶10 and the other two at 1∶100 concentration, as well as two control mixtures containing all three odorants at either 1∶10 or 1∶100 concentrations (Table 6). All scent mixtures and odorants tested (5µl) were applied to 1×0.5cm filter paper strips, which were transferred to 1ml syringes. 1M sucrose solution was used throughout as reward.

### Experimental procedure

One bee at a time was placed into the constant airstream produced by the stimulus controller, and left for 10 s to familiarise itself with the experimental context. After 10 s the training odour was presented to the bee for 6 s. Three sec after onset of the training odour, the antennae were touched with a droplet of sucrose solution exuding from the needle of a syringe, leading to extension of the proboscis. The bee was allowed to feed for 3 s, removed from the airstream, and the next bee was trained. Inter-trial intervals of 10 min were used throughout. Bees were trained to odorants or odour mixtures (see below) four times over two days. This procedure, where all bees were kept under controlled laboratory conditions for two days was used in order to standardize the bees as much as possible. Three trials were conducted on the first day, at the end of which bees were fed to satiation and left in the humidor overnight; a fourth trial was conducted on the morning of the second day, both to test whether the bees had formed a robust and stable memory of the training odour, and to reinforce the olfactory memory. Experiments investigating odorant learning concluded at this stage. In the other experiments, an unrewarded test odour was then presented to the bee for 6 s, and her response recorded. Then the training odour was again presented and rewarded as above, before the next unrewarded test odour was presented, and so on. The sequence of the unrewarded test odours was random for each bee. Interspersing the unrewarded test trials with a rewarded training trial prevented extinction as is likely to occur with repeated unrewarded odour presentation, and is also a way of controlling for the level of responsiveness. Importantly, during these interspersed training trials, the bees were only allowed to feed for 0.5–1s, to prevent overfeeding, which would result in reduced responsiveness. Only bees that always extended the proboscis to the training stimulus throughout the duration of a trial were used for the analysis, on average 20–28 per trial out of 30 bees. Bees that were over responsive, i.e. responded with PER to the airstream, the view of the syringe or to the solvent controls (hexane and ethanol) were excluded, as well as bees that were under responsive, i.e. did not even extend their proboscis after sugar stimulation on the antennae. For each trial of 30 bees, on average 2–5 bees fell into the categories over-responsive or under-responsive and were thus excluded.

### Odorant Learning Trials

Thirty-two groups of 30 bees each were trained to the 32 floral odorants (one group trained to one odorant) over two days, with three acquisition trials on day one and a fourth reinforcement trial on day two. Efficiency of learning was compared across odorants.

### Complex Scent Trials

A group of 30 bees was trained with the first of the complex mixtures, composed of 14 odorants (Table 2). Three training trials with the complex mixture were conducted on day one, followed by a fourth reinforcement trial on day two. The trained bees were then presented with each of the 14 single odorants (unrewarded), one after the other in random sequence, interspersed by rewarded training trials using the complex mixture. The experiment was repeated with a fresh group of thirty bees and the second complex scent mixture, and then a third group of bees using the third scent mixture. Data were analysed separately for each group comparing responses to single odorants with response to the respective training mixture. Based on this analysis, six new scent mixtures (Table 2) were made using either the odorants to which the bees had responded well (key odorant mixtures), or the odorants to which bees had not responded well (non-key-odorant mixtures). A new group of 30 bees was again trained to the first of the original complex scent mixtures of 14 odorants four times over two days as above, and then tested with the corresponding new mixture composed of the key odorants, followed by the mixture composed of the non-key-odorants. This was repeated with the second and third complex scent mixtures and their corresponding key odorant and non-key-odorant mixtures, using new groups of bees for each experiment. Responses to the 14-odorant mixtures were compared with responses to the corresponding key odorant and non-key-odorant mixtures. To investigate discrimination of the three complex mixtures, another group of 30 bees was trained four times to the first 14-odorant mixture as above, and then tested with the second and third 14-odorant mixture (unrewarded), interspersed by a rewarded trial with the first mixture. This was repeated by training a new group of bees to the second mixture and testing them with mixture one and three, and training a third group of bees to the third mixture and testing them with mixture one and two.

### Odorant Uniqueness Trials

A group of 30 bees was trained to a mixture of three odorants, with one of the odorants having a different functional group than the other two: β-pinene, 1-butanol, and 1-hexanol (Table 6). Bees were trained as above four times over two days with the mixture, and then tested with the three single odorants (unrewarded) and an unrewarded control odorant (myrcene), interspersed by rewarded training trials with the mixture. The control odorant was not part of the training mixture, but had the same functional group/chemical characteristic as the unique odorant (β-pinene). The experiment was repeated with another group of bees using the second mixture of three odorants (benzaldehyde, 2-hexanone, and 6-methyl-5-hepten-2-one; control odorant: butanal), and a third group of bees using the last mixture (ethylacetate, benzylalcohol, and 2-phenylethanol; control odorant: ethylhexanoate) (Table 6).

### Odorant Concentration Trials

Three groups of 30 bees each were trained four times over two days as above, to limonene at 1∶10, 1∶100, and 1∶1000, to establish the acquisition efficiency for this odorant at different concentrations. This was repeated with new groups of bees using myrcene and β-pinene as odorants. To test whether they could discriminate the three fairly similar floral odorants at all three concentrations, concentration-dependent discrimination trials were run. A group of bees was trained four times as above to limonene (1∶1000), and then tested with myrcene (1∶1000, unrewarded), and β-pinene (1∶1000, unrewarded), interspersed by a training trial with limonene. The experiment was repeated using the training odorant limonene at 1∶100, as well as the test odorants myrcene and β-pinene at 1∶100. It was repeated a third time using the 1∶10 concentrations of limonene and the testing odorants. The entire set was then repeated using myrcene as training odorant (limonene and β-pinene as test odorants), and then β-pinene as training odorant (limonene and myrcene as test odorants). For each set a new group of bees was used.

Finally a group of bees was trained as above to a mixture of limonene, myrcene and β-pinene, with limonene at 1∶10 and the other two odorants at 1∶100 concentration (Table 6). Bees trained in this way were then tested with the single unrewarded odorants first at 1∶1000, then at 1∶100, finally at 1∶10 concentrations, always interspersed with a rewarded training trial using the mixture. The experiment was repeated with a second group of bees trained to the same mixture of odorants, but with myrcene at 1∶10, and limonene and β-pinene at 1∶100 concentration, and with a third group of bees trained to the same mixture, but with β-pinene at 1∶10, and limonene and myrcene at 1∶100 concentration (Table 6). Two last groups of bees were trained and tested as above using two control mixtures, which contained the three odorants either all at 1∶100 or all at 1∶10 concentration (Table 6).

### Data Analysis and Statistics

During all experiments, the response to the presented odour was recorded, that is, whether the bees extended their proboscis after the onset of the odour and before the presentation of the sucrose reward, in case of the rewarded training trials. This response was recorded as positive PER: bees responded to the presented odour based on the associative memory they had formed. If the bees did not respond to odour presentation in case of the unrewarded testing odours, it was recorded as negative. The percentages of positive PER recorded during odorant acquisition were used to plot learning curves (see Figure 1). To test whether bees had learnt the odorants in a similar way (efficiency of learning), General Linear Model Analysis of Variance (GLM ANOVA) was used, which allows testing of unbalanced models with unequal cell frequencies.

In the complex scent trials, odorant uniqueness trials, and odorant concentration trials Cochran's Q test for overall heterogeneity of the data followed by individual McNemar's Chi squared tests with continuity correction [2×2 table] were applied to analyse responses of bees to unrewarded odorants and test mixtures. To reduce the risk of type I errors due to the multiple use of the same data, we corrected the significance thresholds using the Bonferroni method for dependent data (α′ = α/k), where k is the number of comparisons. P-values between α′ and 0.05 were considered as near significant.

## Supporting Information

Figure S1 Honeybee response to odorants learnt as part of a mixture vs learnt alone
(0.12 MB PDF)

Table S1 Acquisition efficiency for odour mixtures
(0.04 MB DOC)

Table S2 Acquisition efficiency of Limonene, Myrcene, and β-Pinene at three different concentrations
(0.04 MB DOC)

Table S3 Discrimination efficiency of Limonene, Myrcene, and β-Pinene at three different concentrations
(0.04 MB DOC)
