# Supplementary material for: Honeybees Learn Odour Mixtures via a Selection of Key Odorants
Source: PLoS One. 2010 Feb 8;5(2):e9110. doi: 10.1371/journal.pone.0009110 (PMC2817008; doi:10.1371/journal.pone.0009110)
Supplement: Figure S1 — Honeybee response to odorants learnt as part of a mixture vs learnt alone (0.12 MB PDF) [file pone.0009110.s001.pdf]

Figure S1

Honeybee response to odorants learnt as part of a mixture vs learnt alone

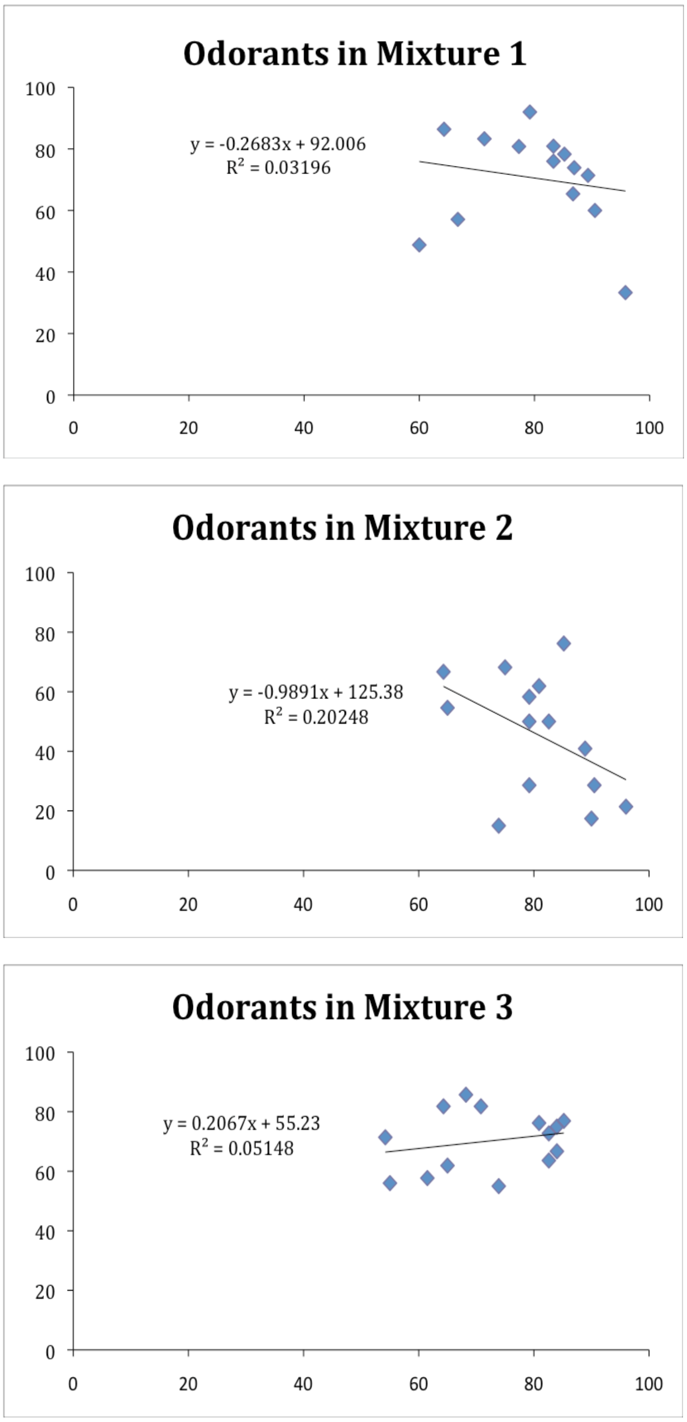

Honeybee response to odorants when learning them as part of a mixture is not correlated to how the odorants are learnt when presented alone. Shown are scatter plots and regression lines for maximum PER response to odorants when tested alone compared to when tested after learning the odorants as part of a mixture. X-axis: PER [%] to odorant when learnt alone; Y-axis: PER [%] to odorant when learnt as part of a mixture. Data taken from Table 4. For odorants and mixture composition see table 1 and Table 2.
