# Supplementary material for: Honeybees Learn Odour Mixtures via a Selection of Key Odorants
Source: PLoS One. 2010 Feb 8;5(2):e9110. doi: 10.1371/journal.pone.0009110 (PMC2817008; doi:10.1371/journal.pone.0009110)
Supplement: Table S1 — Acquisition efficiency for odour mixtures (0.04 MB DOC) [file pone.0009110.s002.doc]

**Table S1.** Acquisition efficiency for odour mixtures

| **Mixture** | **Trial 1** | **Trial 2** | **Trial 3** | **Trial 4** | ***No. Bees*** |
| --- | --- | --- | --- | --- | --- |
| Mix 1 | 0 | 79.8 | 87.1 | 92.7 | *28* |
| Mix 2 | 0 | 84.0 | 89.8 | 85.9 | *27* |
| Mix 3 | 0 | 67.6 | 78.9 | 83.9 | *28* |
| Mix 4 | 0 | 58.3 | 79.0 | 91.1 | *29* |
| Mix 5 | 0 | 66.5 | 85.3 | 93.5 | *27* |
| Mix 6 | 0 | 64.2 | 90.3 | 95.7 | *28* |
| Mix 7 | 0 | 66.7 | 89.7 | 100.0 | *28* |
| Mix 8 | 0 | 70.0 | 90.0 | 100.0 | *26* |
| Mix 9 | 0 | 63.3 | 83.3 | 93.3 | *27* |
| Mix 10 | 0 | 64.3 | 89.7 | 100.0 | *28* |
| Mix 11 | 0 | 59.3 | 85.7 | 100.0 | *27* |

Given are the percentages of maximum Proboscis-Extension-Reflex (PER) response to each mixture over four consecutive trials. For mixture composition see Table 2.
