# Supplementary material for: Honeybees Learn Odour Mixtures via a Selection of Key Odorants
Source: PLoS One. 2010 Feb 8;5(2):e9110. doi: 10.1371/journal.pone.0009110 (PMC2817008; doi:10.1371/journal.pone.0009110)
Supplement: Table S2 — Acquisition efficiency of Limonene, Myrcene, and β-Pinene at three different concentrations (0.04 MB DOC) [file pone.0009110.s003.doc]

**Table S2.** Acquisition efficiency of Limonene, Myrcene, and β-Pinene at three different concentrations

| **Odorant** | **Concentration** | **Trial 1** | **Trial 2** | **Trial 3** | **Trial 4** | ***No. Bees*** |
| --- | --- | --- | --- | --- | --- | --- |
| Limonene | 1:10 | 0 | 50.0 | 83.3 | 86.7 | *27* |
|  | 1:100 | 0 | 43.3 | 81.3 | 83.3 | *30* |
|  | 1:1000 | 0 | 33.3 | 66.7 | 86.7 | *28* |
| Myrcene  | 1:10 | 0 | 57.1 | 81.5 | 80.3 | *24* |
|  | 1:100 | 0 | 27.6 | 65.5 | 81.2 | *29* |
|  | 1:1000 | 0 | 18.5 | 40.7 | 80.2 | *28* |
| β-Pinene | 1:10 | 0 | 83.8 | 87.5 | 87.5 | *28* |
|  | 1:100 | 0 | 48.3 | 72.4 | 83.1 | *29* |
|  | 1:1000 | 0 | 25.0 | 70.0 | 87.5 | *27* |

The odorants are listed alphabetically. Given are the percentages of Proboscis-Extension-Reflex (PER) response to each odorant over four consecutive trials.
