# Supplementary material for: Honeybees Learn Odour Mixtures via a Selection of Key Odorants
Source: PLoS One. 2010 Feb 8;5(2):e9110. doi: 10.1371/journal.pone.0009110 (PMC2817008; doi:10.1371/journal.pone.0009110)
Supplement: Table S3 — Discrimination efficiency of Limonene, Myrcene, and β-Pinene at three different concentrations (0.04 MB DOC) [file pone.0009110.s004.doc]

**Table S3.** Discrimination efficiency of Limonene, Myrcene, and β-Pinene at three different concentrations

| **Training Odorants** |  | **Testing Odorants** |  |
| --- | --- | --- | --- |
|  | Limonene 1:10 | Myrcene 1:10 | β-Pinene 1:10 |
| Limonene 1:10 | - | 14.3 | 7.1 |
| Myrcene 1:10 | 16.7 | - | 4.2 |
| β-Pinene 1:10 | 10.3 | 6.9 | - |
| **Training Odorants** |  | **Testing Odorants** |  |
|  | Limonene 1:100 | Myrcene 1:100 | β-Pinene 1:100 |
| Limonene 1:100 | - | 20.7 | 17.2 |
| Myrcene 1:100 | 21.4 | - | 14.3 |
| β-Pinene 1:100 | 14.3 | 17.9 | - |
| **Training Odorants** |  | **Testing Odorants** |  |
|  | Limonene 1:1000 | Myrcene 1:1000 | β-Pinene 1:1000 |
| Limonene 1:1000 | - | 29.6 | 22.2 |
| Myrcene 1:1000 | 32.4 | - | 21.4 |
| β-Pinene 1:1000 | 25.9 | 22.2 | - |

The odorants are listed alphabetically. Given are the percentages of Proboscis-Extension-Reflex (PER) response to the test odorants (*N*=27-29 bees for each trial).
